# Pathological characteristics and clinical prognostic analysis of intravenous leiomyomatosis: a retrospective study of 43 cases

**DOI:** 10.3389/fmed.2025.1534933

**Published:** 2025-04-10

**Authors:** Jiezhen Li, Haijian Huang, Xin Chen, Qiang Zeng

**Affiliations:** ^1^Department of Pathology, Fujian Provincial Hospital, Provincial Clinical Medical College of Fujian Medical University, Fuzhou, China; ^2^Department of Pathology, The First Affiliated Hospital of Fujian Medical University, Fuzhou, China

**Keywords:** intravenous leiomyomatosis, cervical tumor, recurrence, histopathology, immunohistochemistry

## Abstract

**Objective:**

To analyze the clinicopathological features and prognostic factors of intravenous Leiomyomatosis (IVL), a rare yet recurrent disease.

**Methods:**

This retrospective observational study enrolled 43 patients with pathologically confirmed IVL. Clinicopathological data were collected and reviewed. Univariate analyses were performed to identify prognostic factors for IVL recurrence.

**Results:**

Clinical manifestations included increased menstrual flow (12/43), prolonged menstrual periods (18/43), pelvic mass (15/43), abdominal pain (8/43), or no symptoms (9/43). Histopathologically, tumor cells were predominantly located in the blood vessels of the uterine muscle wall and surrounding blood vessels. The tumor were composed of benign smooth muscle cells arranged in strips or bundles. Immunohistochemistry revealed that tumor cells were positive for SMA (43/43), Desmin (42/43), and Caldesmon (40/43). Incomplete resection of the lesions was identified as a risk factor for postoperative recurrence of IVL (*P* < 0.05). Age, menopause status, gravidity, parity, maximum diameter of IVL, uterine leiomyomas, involvement of uterine/extrauterine blood vessels, surgical methods, Ki-67 index, and mitotic figures were not associated with postoperative recurrence of IVL (*P* > 0.05).

**Conclusion:**

IVL is a rare form of leiomyoma with potential for malignancy. Complete resection of the lesion should be performed whenever possible to improve patient prognosis.

## Introduction

Intravenous leiomyomatosis (IVL) is a rare and special type of leiomyoma, with only over 400 cases reported worldwide (1–10). The histopathological morphology of IVL is similar to that of ordinary leiomyomas; however, its biological behavior is malignant (1, 2). It can spread along the uterine or ovarian veins to the inferior vena cava, right atrium, and pulmonary artery (3–5). Early IVL lacks specific clinical symptoms and characteristic imaging manifestations, making preoperative diagnosis difficult. Pathologically, IVL intersects with the morphology and immunophenotype of ordinary leiomyomas, leiomyosarcomas, and low-grade endometrial stromal tumors, making it prone to misdiagnosis. The overall prognosis of IVL is good; however, some cases recur within months to years at a rate of 16.8%–30.0% (6–10). There is limited research on the factors affecting recurrence and the pathological characteristics of IVL. This study aimed to explore the clinical, pathological, and prognostic features of IVL by evaluating 43 cases from two provincial-level comprehensive hospitals in China.

## Materials and methods

### Patient selection and case review

We retrospectively collected the clinical and pathological data of 43 patients with IVL diagnosed at the Pathology Department of Fujian Provincial Hospital and the First Affiliated Hospital of Fujian Medical University from August 2011 to August 2023. All patients were diagnosed by three female reproductive system pathology specialists. The tissue samples obtained from patients were fixed in 10% neutral formalin solution (pH 7.2) for 24 h, dehydrated routinely, embedded in paraffin, sectioned at 4 μm, stained with hematoxylin and eosin, and observed. This trial was approved by the ethics committee of the hospital (approval number: K2024-01-012), and patients voluntarily participated and provided written informed consent. Initial operation type, demographic information, and clinical data were obtained from the electronic case system.

### Immunohistochemical evaluation

Immunohistochemical staining was performed using the En-Vision two-step method. All antibodies came from Fuzhou Maixin Biotechnology Co., Ltd. in China. The following antibodies were used in this experiment: SMA (MX097), Desmin (MX046), Caldesmon (h-CALD), ER (SP1), PR (SP2), CD10 (MX002), P16 (MX007), CyclinD1 (SP4), SMARB1(MRQ-27), FH (J-13), SDHB (MX096), CD31 (MX032), CD34 (QBEnd/10), and KI67(MX006). The degree of staining was evaluated using a semiquantitative scoring method for IVL and surrounding non-neoplastic tissue: 0 (no staining), 1 (<5%), 2 (5%–24%), 3 (25%–50%), and 4 (>50%), and the means of the two were compared by Student’s *t*-test.

### Follow-up and prognostic observations

All patients underwent regular follow-up every 6–9 months postoperatively. The deadline for follow-up was 31 August 2023. The endpoint of this study was tumor recurrence. Recurrence was defined as the detection of a mass with a diameter of >1 cm at the same location during two consecutive imaging examinations.

### Statistical analysis

Microsoft Excel was used to screen, classify, and summarize the data. Statistical analyses were performed using SPSS for Windows 22 (IBM, Armonk, NY). Chi-square test was used to analyze the correlation between clinical pathological features and postoperative recurrence of IVL. Kaplan–Meier survival analysis and log-rank test were used to verify the correlation between recurrence and various factors. *P* < 0.05 was considered statistically significant.

## Results

### Clinicopathological characteristics of IVL

#### Clinical characteristics

**FIGURE 1:**
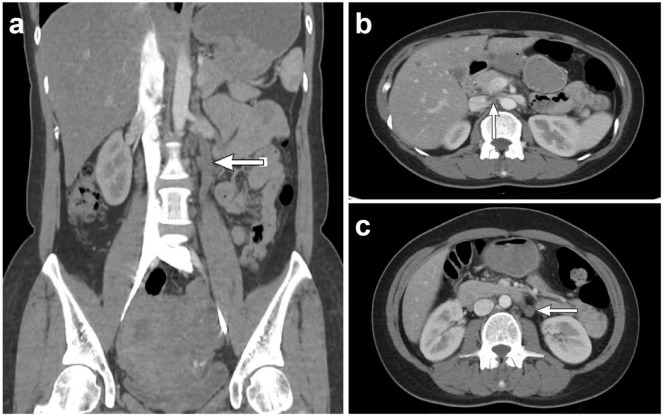
Computed tomography scan shows that when intravenous leiomyomatosis involves the parametrial/extrauterine blood vessels, thickening of the venous wall can be observed, accompanied by a cord-like filling defect [**(a)** left ovarian vein filling defect; **(b)** left renal vein and inferior vena cava filling defect; and **(c)** left ovarian vein filling defect].

The study included 43 patients aged 15–62 years (median: 45 years), with 10 and 33 menopausal and premenopausal patients, respectively. The clinical manifestations were increased menstrual flow (12/43), prolonged menstrual period (18/43), pelvic mass (15/43), abdominal pain (8/43), or no symptoms (9/43) (Table 1). Ultrasound revealed an uneven distribution of echogenic light spots in the uterine myometrium, with multiple low echogenicities inside. Multiple oval- and cord-shaped hypoechoic lesions with abundant blood flow signals can be detected in patients with periuterine/extrauterine blood vessels. Abdominal CT revealed multiple nodular and blocky low-density shadows in the uterine muscle wall with uneven enhancement after enhancement. For tumors involving periuterine/extrauterine blood vessels, thickening of the venous wall was observed, accompanied by a cord-like filling defect (Figure 1). In most cases, preoperative diagnosis was challenging, and the preoperative diagnostic rate in this group was 27.9% (12/43). All patients (*n* = 43) underwent surgical resection, namely myomectomy (*n* = 5), total hysterectomy (*n* = 7), and total hysterectomy with fallopian tubes (*n* = 31) (Table 1). Oophorectomy was performed in 16 cases, while 27 patients did not undergo oophorectomy.

**TABLE 1:** Overall characteristics of 43 patients with intravenous leiomyomatosis.

Patient characteristics	No. of patients (*n* = 43)	Percentage (%)
Age range (years)	15–62 (median age 45)	–
**Presenting symptoms**		
Increased menstrual flow	12	27.9
Prolonged menstrual period	18	41.9
Pelvic mass	15	34.9
Abdominal pain	8	18.6
No symptoms	9	20.9
Menopause status	10	23.3
**Gravidity**		
≥2	30	69.8
**Parity**		
≥2	14	32.6
Involvement of intrauterine/extrauterine vessels	13	30.2
Combined uterine fibroids	29	67.4
**Operation type**		
Myomectomy	5	11.6
TH	7	16.3
TH-BS	31	72.1
Complete resection	36	83.7
Ovariectomy	16	37.2
**Ki-67 index**		
≥5%	9	20.9
**Mitotic figures**		
≥2	6	14.0

TH, total hysterectomy; BS, bilateral salpingectomy.

#### Intraoperative findings

During surgery, white cord-like masses were observed in 19 patients, yellowish-white worm-like masses in 15, yellow mucoid masses in 3, and white solid nodular masses in 6. In 13 patients (30.2%), periuterine/extrauterine blood vessels, including the vaginal veins, broad ligament veins, uterine veins, iliac veins, renal veins, and inferior vena cava, were affected (Table 2). Intraoperative examination revealed the thickening and cord-like appearance of the broad ligament of the uterus and the affected extrauterine large veins, exhibiting a solid consistency upon palpation with no palpable pulsation. Among the 43 patients who underwent surgery, complications were noted, which included 29, 5, and 3 cases of uterine fibroids, adenomyosis, and endometrial polyps, respectively.

**TABLE 2:** Clinical and pathological data of 13 cases of intravenous leiomyomatosis involving intrauterine/extrauterine vessels.

Case	Age (years)	Menopause	G and P	Presenting symptoms	Maximum diameter (cm)	Combined uterine fibroids	Operation type	Ovariectomy	Complete resection	Ki-67 (%)	Mitotic figures/ 10HPF	Recurrence/ time (m)
1	45	No	G1P1	PMP, IMF	6.5	No	TH-BS	Yes	Yes	15	<2	No/4
2	46	Yes	G3P1	Abdominal pain, pelvic mass, PMP	17.8	No	TH-BS	No	No	3	<2	Yes/30
3	53	Yes	G3P1	PMP, IMF	12.0	Yes	TH-BS	Yes	Yes	2	<2	No/42
4	43	No	G5P2	Pelvic mass, abdominal pain	15.0	No	TH-BS	No	No	2	<2	No/39
5	48	Yes	G4P1	No symptoms	6.8	Yes	TH-BS	Yes	Yes	5	<2	Yes/89
6	39	No	G1P1	Pelvic mass, IMF	5.0	Yes	TH	No	Yes	1	<2	No/14
7	47	No	G1P1	PMP, IMF	28.5	No	TH	No	No	10	3∼4	Yes/99
8	47	No	G3P3	PMP	7.5	Yes	TH-BS	No	Yes	1	<2	No/36
9	42	No	G2P1	IMF	6.0	No	TH-BS	No	Yes	1	<2	No/77
10	39	No	G1P1	Pelvic mass	5.8	Yes	TH-BS	No	Yes	1	<2	No/68
11	40	No	G1P1	IMF, abdominal pain	11.0	Yes	TH-BS	No	Yes	2	<2	No/10
12	45	No	G3P1	PMP	15.0	Yes	TH-BS	Yes	Yes	15	<2	No/65
13	45	No	G1P1	PMP	9	Yes	TH-BS	No	No	1	<2	No/6

G and P, gravidity and parity; TH, total hysterectomy; BS, bilateral salpingectomy; HPF, high power field; m, months; PMP, prolonged menstrual period; IMF, increased menstrual flow.

#### Macroscopic examination

**FIGURE 2:**
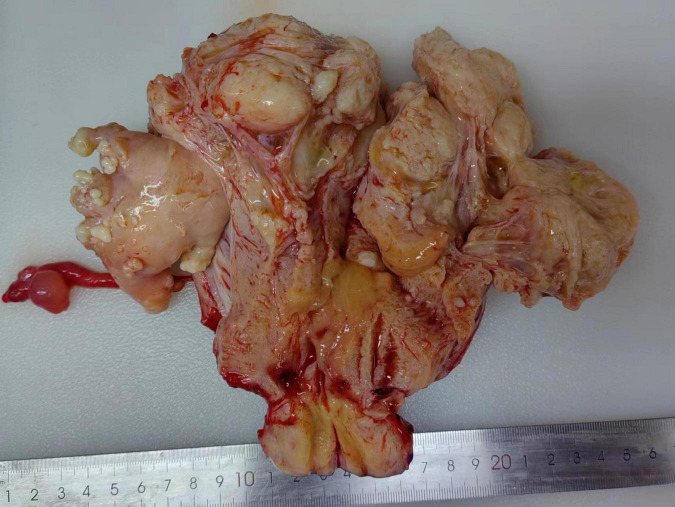
Nodular, linear, and worm-like masses can be seen in the uterine muscle wall, which are easily pulled out and appear in a bead-like shape, with varying thicknesses.

The masses between the uterine muscle walls and in the affected blood vessels are smooth, cord-like, worm-like, and nodular in shape, which are easy to pull out and vary in thickness (Figure 2). The surface is smooth, and the cut surface is gray and white, with a tough texture. The maximum diameter of IVL ranged 1.5–32.0 cm (median: 7.0 cm). Among the 43 patients with IVL, 29 cases (67.4%) were complicated with common leiomyoma.

#### Microscopic examination

**FIGURE 3:**
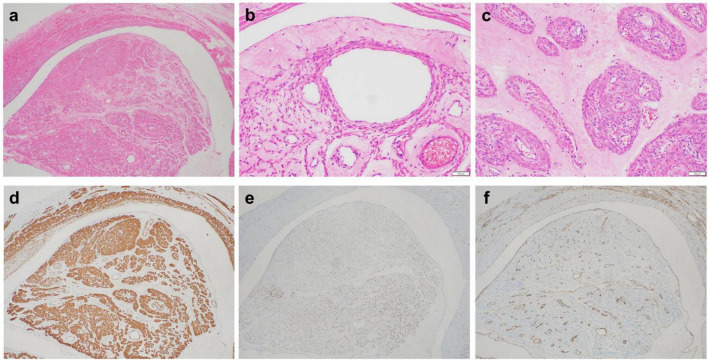
**(a)** The tumor is mainly located in the veins, which can present as a polyp-like protrusion filling the vascular lumen. **(b)** The tumor stroma is significantly loose and edematous, and the edematous stroma divides the tumor cells into single linear strips. Endothelial cells line the surface of the tumor. **(c)** The loose and edematous stroma divides tumor cells into nested pieces. Tumor cells have a mild morphology and eosinophilic cytoplasm. Tumor stroma is rich in thick-walled small blood vessels. **(d)** Tumor cells expressing SMA. **(e)** Tumor cells expressing ER. **(f)** The surface of the tumor expresses CD34, whereas CD34 outlines the tumor stroma rich in blood vessels.

The tumor is mainly located in the veins and is present as a polyp-like protrusion toward or filling the vascular lumen (Figure 3a). Fissure formation can be observed between tumor cells and vascular endothelial cells. The surface of the tumor is lined with endothelial cells. The tumor is mainly composed of warm and smooth muscle cells arranged in bundles, cords, or nodules. Cell density varied, with six cases having abundant tumor cells and 37 with moderate cell density. The tumor has interstitial edema with vitreous degeneration. The edematous stroma divides tumor cells into a single linear strip shape (Figure 3b) and a nested sheet shape (Figure 3c). The tumor stroma is rich in blood vessels, and the vascular density is higher inside the tumor than that inside the surrounding normal uterine smooth muscle tissue, forming a sponge-like vascular network inside the tumor. No cases of necrosis were observed. Mitotic figures in 2–3/10 HPF were observed in six cases (6/43), whereas the remaining 37 cases (37/43) had mitotic figures <2/10 HPF.

#### Immunophenotype

Tumor cells diffusely expressed SMA (43/43) (Figure 3d), Desmin (42/43), Caldesmon (40/43), ER (35/43) (Figure 3e), and PR (32/43). Partial tumor cells expressed CD10 (3/43), P16 (7/43), and CyclinD1 (5/43). All IVLs retained the expression of SMARB1, FH, and SDHB. Endothelial cells and vascular endothelial cells on the tumor surface express CD31 and CD34 (Figure 3f), with a Ki-67 proliferation index of <5% and ≥5% in 34 and 9 patients, respectively.

### Analysis of postoperative recurrence factors in patients with IVL

#### Postoperative recurrence

In total, 43 patients with IVL were followed up for 4–137 months, with a median follow-up time of 40 months. During the follow-up period, no deaths were reported, and recurrence was observed in 11.6% of the patients (5/43), with a recurrence interval of 30–89 (median time, 78) months (Table 3). The five patients who experienced a recurrence were aged 15–48 years (median age, 46 years). Three of these patients underwent total hysterectomy and salpingectomy, of which two had a recurrence at the vaginal stump (one patient was monitored without surgical treatment after recurrence, while the other underwent myomectomy and is currently tumor-free), and one experienced a recurrence within the abdominal cavity (the patient underwent myomectomy after recurrence and is currently tumor-free). Moreover, of the five patients, one underwent total hysterectomy and had a recurrence located near the retroperitoneum in the abdominal cavity; after myomectomy, this patient is also tumor-free. Another patient underwent hysteroscopic myomectomy and had a recurrence in the uterus. Because of her young age at the time of recurrence (22 years old), she underwent another myomectomy and remains tumor-free as well.

**TABLE 3:** Clinical and pathological data of five recurrent cases of intravenous leiomyomatosis.

Case	Age (years)	Presenting symptoms	Maximum diameter (cm)	Combined uterine fibroids	Opera-tion type	Ovariec-tomy	Complete resection	Uterine/ extrau-terine vessels	Ki-67 (%)	Mitotic figures/ 10HPF	Recurr-ence interval (m)	Re-current site	Manifesta-tions after recurrence	Treatment after recurrence
1	15	Abdominal pain	6.5	Yes	Myomectomy	No	No	No	2	<2	78	Uterus	PMP	Myomectomy
2	36	Pelvic mass, PMP	12.0	No	TH-BS	Yes	Yes	No	10	3∼4	46	Abdominal cavity	Abdominal pain	Myomectomy
3	46	Abdominal pain, pelvic mass, PMP	17.8	No	TH-BS	No	No	Yes	3	<2	30	Vaginal stump	Pelvic mass (detected on CT scan)	Myomectomy
4	47	PMP, IMF	28.5	No	TH	No	No	Yes	10	3∼4	99	Abdominal cavity	Abdominal pain	Myomectomy
5	48	No symptoms	6.8	Yes	TH-BS	Yes	Yes	Yes	5	<2	89	Vaginal stump	No symptoms (detected by ultrasound examination)	Observation and conservative treatment

BS, bilateral salpingectomy; HPF, high power field; IMF, increased menstrual flow; m, months; PMP, prolonged menstrual period; TH, total hysterectomy.

#### Analysis of recurrence factors

**FIGURE 4:**
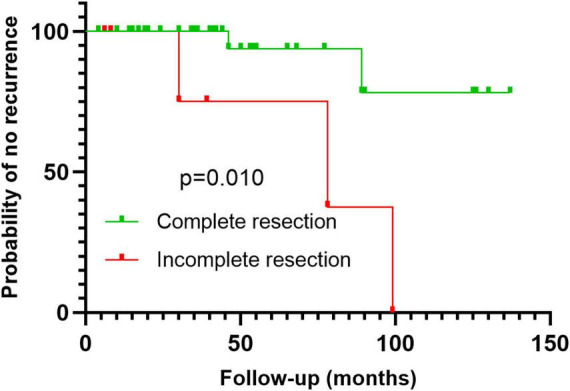
Kaplan–Meier survival analysis with log-rank test shows that incomplete resection is a risk factor for postoperative recurrence.

The correlation analysis between clinical pathological features and recurrence risk is summarized in Table 4. Analysis of postoperative recurrence factors showed that incomplete resection of lesions was a risk factor for postoperative recurrence of IVL (*P* < 0.05) (Table 4). Age, menopause, gravidity, parity, maximum diameter of IVL, uterine leiomyomas, involvement of uterine/extrauterine blood vessels, methods of operation, ovariectomy or not, Ki-67 index, and mitotic figures were not associated with postoperative recurrence of IVL (*P* > 0.05) (Table 4). Kaplan–Meier survival analysis was used to evaluate the correlation between recurrence and related factors, and similar results were obtained (Figure 4).

**TABLE 4:** Analysis of postoperative recurrence factors for intravenous leiomyomatosis.

	Recurrence (*n* = 5)	Nonrecurrence (*n* = 38)	*P*
Age (years)			1.000
<45	2(40.0)	15(39.5)	
≥45	3(60.0)	23(60.5)	
Maximum diameter (cm)			1.000
<7	2(40.0)	18(47.4)	
≥7	3(60.0)	20(52.6)	
Menopause			0.575
Yes	2(40.0)	8(21.1)	
No	3(60.0)	30(78.9)	
Gravidity			0.630
< 2	2(40.0)	11(28.9)	
≥2	3(60.0)	27(71.1)	
Parity			1.000
<2	4(80.0)	25(65.8)	
≥2	1(20.0)	13(34.2)	
Uterine/extrauterine vessels			0.153
Yes	3(60.0)	10(26.3)	
No	2(40.0)	28(73.7)	
Uterine leiomyomas			0.309
Yes	2(40.0)	27(71.1)	
No	3(60.0)	11(28.9)	
Operation type			0.595
Myomectomy	1(20.0)	4(10.5)	
TH	1(20.0)	6(15.8)	
TH-BS	3(60.0)	28(73.7)	
Complete resection			0.024
Yes	2(40.0)	34(89.5)	
No	3(60.0)	4(10.5)	
Ovariectomy			1.000
Yes	2(40.0)	14(36.8)	
No	3(60.0)	24(63.2)	
Ki-67 index			0.054
<5%	2(40.0)	32(84.2)	
≥5%	3(60.0)	6(15.8)	
Mitotic figures			0.135
<2	3(60.0)	34(89.5)	
≥2	2(40.0)	4(10.5)	

TH, total hysterectomy; BS, bilateral salpingectomy.

## Discussion

This study included 43 cases from two provincial-level comprehensive hospitals in China, which were carefully evaluated based on their clinical and pathological characteristics, immunohistochemical features, and postoperative recurrence, thus representing one of the main series. The median age for IVL diagnosis is 46 years (range 21–81) (6, 8). Consistent with the literature, patients in this study were aged 15–62 years (median: 45 years), and the 15-year-old patient in this study is currently the youngest reported patient. The clinical manifestations of IVL are diverse, appearing mainly as abdominal masses (54.6%), followed by asymptomatic masses (21.5%), excessive menstrual flow (11.5%), prolonged menstrual cycle (6.2%), and pelvic pain (6.2%). Approximately 15.0% of patients had a history of uterine fibroidectomy (6). Imaging can aid in the preoperative diagnosis of some typical IVL, but its diagnostic rate is relatively low. In particular, tumors with a small volume and those limited to the uterine muscle wall have nonspecific imaging manifestations; these are easily misdiagnosed as ordinary leiomyomas (10, 11). The preoperative diagnostic rate of IVL reported by Tang and Lu (10) was 3/13 (23.1%), which was similar to our study, with a rate of 12/43 (27.9%). Pathological examination is essential for IVL to avoid misdiagnosis.

Macroscopic examination of IVL is specific. IVL forms nodular, cord-like, and worm-like tumor emboli between the uterine muscle walls (2, 10). The histopathology of IVL is similar to that of ordinary leiomyomas, arranged in bundles, cords, or nodules, with mild cell morphology (11, 12). However, IVL tumor cells grow inside the blood vessels unlike ordinary leiomyomas. Our study found that IVL tumors have higher microvascular density than the surrounding normal uterine smooth muscle tissue and that a sponge-like vascular network is formed inside the tumor. This indicates that IVL may have a strong inducing effect on the proliferation of vascular endothelial cells and neovascularization. Unlike ordinary leiomyomas, IVL tumors have more pronounced and widespread interstitial edema and hyaline degeneration. Immunophenotypically, IVL tumor cells express smooth muscle-derived markers (SMA, Desmin, and Caldesmon) and ER and PR. Partial IVL cases show varying degrees of expression of P16 and CyclinD1 (10–12).

Currently, little information on the molecular pathological mechanisms involved in this feature is available, and only a few reports are available on the molecular genetics of IVL (1, 12–16). IVL has multiple chromosomal aberrations, with the most common being 22q11.23-q13.31 and 22q12.3-q13.1 deletions. IVL develops because of deletions in the 22q and 1p chromosomal regions and additions in the 12q chromosomal region; these changes overlap with the chromosomal changes observed in uterine leiomyosarcoma (14). This finding further supports the biological behavior of IVL, which displays intermediate and malignant potential.

Ordulu et al. (1) identified overexpression of HMGA2 in 58% (7/12) of IVL cases through fluorescence *in situ* hybridization analysis. HMGA2 serves as a driver of tumor metastasis, and its overexpression is an early event in leiomyoma development, which plays a pivotal role in the pathogenesis of IVL. Ura et al. (17) reported the involvement of phosphoproteins, such as HSPB1, HPAS5, HSPD1, and PRDX2, in inhibiting leiomyoma cell apoptosis and promoting cell survival, thereby regulating leiomyoma growth. Excessive phosphorylation of Rb can promote tumorigenesis by blocking apoptosis and stimulating proliferation and invasion. This regulatory pathway has been observed in various tumors. In another study, Ordulu et al. (12) found that all IVL cases displayed cytoplasmic phosphorylated Rb localization. IVL shares certain genetic characteristics with conventional leiomyomas, particularly the presence of the der(14)t(12;14)(q15;q24) karyotype, which is associated with the t(12;14)(q15;q24) genetic subgroup of conventional leiomyomas. However, unlike conventional leiomyomas, mutations in *MED12* are rarely observed in IVL, while *MED12* mutations are the most common in conventional leiomyomas (15, 16). Lu et al. (16) also observed a high frequency of loss of heterozygosity and increased genetic instability in IVL, which is markedly different from conventional leiomyomas. These studies collectively indicate that IVL is a unique tumor entity that must be distinguished from other tumors in routine practice. However, we were unable to conduct genetic testing in this study because of limited genetic testing conditions.

According to the WHO classification, the ICD-O code of IVL is 1 (uncertain malignant potential), and it displays a biological behavior similar to that of malignant tumors, which are prone to recurrence and metastasis. After the initial surgery, IVL can recur within months to years, with a reported recurrence rate of 16.8%–30.0% (6, 7). Peng et al. (6) analyzed 166 patients with a median follow-up period of 36 months, reporting a recurrence rate of 8.4% (14/166), which is lower than that in other studies. IVL mainly recurs in the pelvic cavity and iliac vein, whereas recurrences in the inferior vena cava and heart are rare. There is currently no consensus on the factors that affect IVL recurrence; however, factors such as age, tumor size, incomplete resection, ovarian preservation, postoperative antiestrogen therapy, and invasion of adjacent blood vessels might be involved (2, 6, 9, 18–20). Interestingly, Shi et al. (21) observed that patients with an accidental diagnosis of IVL had a higher recurrence rate than those without an accidental diagnosis. This may be due to insufficient preoperative evaluation as most patients with an accidental diagnosis of IVL were only operated on by gynecologists, resulting in residual lesions and tumor recurrence. Yu et al. (19) reported that the involvement of the large vein is associated with an increased risk of postoperative recurrence. In this study, incomplete resection of lesions was identified as a risk factor for recurrence. Wen et al. (22) conducted an analysis of 216 patients with intravascular leiomyomatosis (IVL), representing the largest single-center report on IVL published to date. Their findings revealed that among patients with completely resected IVL, the recurrence rate during follow-up was 9.7%, whereas for those with incompletely resected IVL, the disease progression/recurrence rate was 39.0%. Furthermore, they discovered that recurrence or progression of residual lesions was associated with postoperative adjuvant aromatase inhibitor therapy and the maximum tumor diameter. Zhang et al. (23) found that incomplete tumor resection, involvement of the iliac/genital veins, involvement of the inferior vena cava, and a tumor diameter of ≥15 cm were independent risk factors for IVL recurrence and progression. Additionally, for patients with IVL involving the iliac/genital veins and proximal veins, even after complete tumor resection combined with bilateral adnexectomy, postoperative adjuvant aromatase inhibitor therapy failed to significantly reduce the recurrence rate. Although the data were collected and analyzed retrospectively, considering the rarity of IVL, a relatively sufficient sample size and longer follow-up period makes our results reliable. The treatment of IVL mainly relies on surgical resection, and the surgical method depends on its size, extent of tumor involvement, and need for preserving reproductive function (24). After complete surgical resection, the patient has a good prognosis and a very low recurrence rate. Therefore, surgeons should aim to remove the lesion as completely as possible. Furthermore, IVL is a sex hormone-dependent tumor that originates in the uterus and exhibits similar sex hormone receptor characteristics to those of the uterine myometrium and uterine leiomyoma. Immunohistochemical staining reveals positive staining for ER and PR. Therefore, exogenous estrogen should be avoided as much as possible after surgery. Studies have found that for patients who retain their uterus and/or ovaries, as well as those with incomplete resection, adjuvant anti-estrogen therapy (such as tamoxifen, aromatase inhibitors, and gonadotropin-releasing hormone agonist GnRH-a) can prevent tumor recurrence or continued growth of residual lesions (25–27). However, some studies have also shown that adjuvant aromatase inhibitor therapy for IVL patients after surgery does not reduce the postoperative recurrence rate (23). Complete resection of the lesion remains the key to preventing IVL recurrence. Recently, Zhang et al. (28) reported on novel targeted therapies, such as the mTOR inhibitor sirolimus, which can safely and effectively treat recurrent IVL. However, its clinical application still needs to be further confirmed through larger clinical studies.

Pathologically, IVL should be distinguished from ordinary leiomyomas, edematous leiomyomas, and smooth muscle sarcomas. Ordinary leiomyoma overlaps with the morphology and immunophenotype of IVL. Macroscopic examination of IVL reveals characteristic cord-like, worm-like, and bead-like tumor thrombi. Microscopically, the special pattern of intravascular growth is helpful for differentiation. Edematous leiomyoma is often arranged in a nodular pattern and is surrounded by edematous connective tissues (29), with no CD34-positive blood vessels surrounding the tumor cells on immunohistochemistry. Leiomyosarcoma commonly has infiltrating boundaries microscopically, and it must be differentiated from IVL when vascular invasion occurs. Notably, leiomyosarcoma has a higher cell density and greater atypia than IVL, and pathological mitosis and tumor necrosis are more common (30).

## Conclusion

In summary, incomplete resection was identified as a risk factor for the postoperative recurrence of IVL. In this study, we focused on describing the histology and immunohistochemistry of IVL. Our findings can provide a basis for the pathological diagnosis, clinical treatment, and prognostication of IVL.

## Data Availability

The original contributions presented in this study are included in this article/supplementary material, further inquiries can be directed to the corresponding author.
